# Exploring Public Emotions on Obesity During the COVID-19 Pandemic Using Sentiment Analysis and Topic Modeling: Cross-Sectional Study

**DOI:** 10.2196/52142

**Published:** 2024-10-11

**Authors:** Jorge César Correia, Sarmad Shaharyar Ahmad, Ahmed Waqas, Hafsa Meraj, Zoltan Pataky

**Affiliations:** 1 Unit of Therapeutic Patient Education WHO Collaborating Center University Hospitals of Geneva and University of Geneva Geneva Switzerland; 2 School of Mathematics, Computer Science & Engineering Liverpool Hope University Liverpool United Kingdom; 3 Department of Primary Care & Mental Health Institute of Population Health University of Liverpool Liverpool United Kingdom; 4 Greater Manchester Mental Health NHS Foundation Trust Salford United Kingdom

**Keywords:** obesity, Twitter, infodemic, attitude, opinion, perception, perspective, obese, weight, overweight, social media, tweet, sentiment, topic modeling, BERT, Bidirectional Encoder Representations from Transformers, NLP, natural language processing, general public, celebrities

## Abstract

**Background:**

Obesity is a chronic, multifactorial, and relapsing disease, affecting people of all ages worldwide, and is directly related to multiple complications. Understanding public attitudes and perceptions toward obesity is essential for developing effective health policies, prevention strategies, and treatment approaches.

**Objective:**

This study investigated the sentiments of the general public, celebrities, and important organizations regarding obesity using social media data, specifically from Twitter (subsequently rebranded as X).

**Methods:**

The study analyzes a dataset of 53,414 tweets related to obesity posted on Twitter during the COVID-19 pandemic, from April 2019 to December 2022. Sentiment analysis was performed using the XLM-RoBERTa-base model, and topic modeling was conducted using the BERTopic library.

**Results:**

The analysis revealed that tweets regarding obesity were predominantly negative. Spikes in Twitter activity correlated with significant political events, such as the exchange of obesity-related comments between US politicians and criticism of the United Kingdom’s obesity campaign. Topic modeling identified 243 clusters representing various obesity-related topics, such as childhood obesity; the US President’s obesity struggle; COVID-19 vaccinations; the UK government’s obesity campaign; body shaming; racism and high obesity rates among Black American people; smoking, substance abuse, and alcohol consumption among people with obesity; environmental risk factors; and surgical treatments.

**Conclusions:**

Twitter serves as a valuable source for understanding obesity-related sentiments and attitudes among the public, celebrities, and influential organizations. Sentiments regarding obesity were predominantly negative. Negative portrayals of obesity by influential politicians and celebrities were shown to contribute to negative public sentiments, which can have adverse effects on public health. It is essential for public figures to be mindful of their impact on public opinion and the potential consequences of their statements.

## Introduction

Obesity is a complex, multifactorial, and relapsing disease that has become increasingly prevalent worldwide among people belonging to all age groups [1]. The age-standardized prevalence of obesity has increased from 4.6% in 1980 to 14% in 2019, with a rising trend globally [1]. It is associated with a high risk of multiple comorbidities, including type 2 diabetes; cardiovascular, respiratory, and digestive diseases; and multiple cancers [2-4]. Obesity also leads to important, negative psychological and social impacts, as well as a significant risk of depression, anxiety disorders, and other psychiatric diseases. Furthermore, obesity is linked to public and internalized stigma, with individuals experiencing low self-esteem, social isolation, and mental health problems [5,6]. People with obesity may also be exposed to bullying, discrimination, and negative stereotypes on social media platforms [7,8].

While primary research, including epidemiological and interventional studies, is essential for understanding and addressing obesity, the importance of public health communication cannot be overstated [9]. Effective health communication can help raise awareness, debunk myths, and promote healthy behaviors related to obesity [10,11]. While social media platforms can be sources of valuable health information, they often contain inaccurate or misleading information, which can be detrimental to public health [7]. The infodemic accompanying the pandemic exemplifies how misleading health information on social media platforms can contribute to fear, unfounded concerns, and poor health behaviors among the general population [9,12]. In addition to providing a platform for the dissemination of potentially harmful misinformation, social media can also perpetuate stereotypes and biases against individuals with obesity, further exacerbating the negative impacts of this condition [7].

Social media platforms (eg, Twitter; subsequently rebranded as X) offer a wealth of information that can be harnessed to gain insights into the dominant health behaviors, attitudes, and sentiments of the masses toward obesity. By analyzing tweets and other social media posts, researchers can examine the perspectives and opinions of the public, celebrities, and influential organizations on this critical health issue [13].

Advanced analytical techniques, such as sentiment analysis and topic modeling, can be used to explore the complex landscape of opinions and emotions surrounding obesity on social media platforms [14]. Sentiment analysis allows for the systematic identification and categorization of subjective information in textual data, providing insights into the prevailing emotions and opinions expressed in social media posts [15]. Topic modeling, on the other hand, is an unsupervised machine learning technique that can uncover hidden patterns and themes within large volumes of text data, revealing the most prominent topics and trends related to obesity in social media discussions [16]. By using these cutting-edge methodologies, this study aims to uncover the dominant narratives, attitudes, and emotions related to obesity on social media platforms. The findings from this analysis will not only provide a comprehensive understanding of public sentiment toward obesity but also inform the development of targeted public health interventions and communication strategies to address this global health challenge.

The research objectives of this study are (1) to examine the role of social media in perpetuating or challenging stereotypes, biases, and stigma against individuals with obesity, and assess the potential impact of these factors on their mental health and well-being; and (2) to identify the most prominent topics and trends in discussions surrounding obesity on social media, examining the content generated by the general public, celebrities, and influential organizations.

## Methods

### Overview

This study uses a combination of sentiment analysis and topic modeling techniques to analyze tweets related to obesity. This approach allowed us to examine both the sentiment and content of the discussions, providing a comprehensive understanding of the perceptions and concerns related to obesity.

### Data Collection

Twitter data were collected using Tweepy, a Python-based open-source wrapper package for the Twitter application programming interface (API) [17]. Tweepy simplifies the communication process with the API and enables access to Twitter’s historical records. Tweepy serves as a reliable, open-source Python package that streamlines communication with the Twitter API, enhancing the overall development experience. We did not apply any restrictions to the country the tweets originated from. However, only tweets in the English language were retrieved. We leveraged Tweepy’s *paginator* function to manage pagination and retrieve all relevant data associated with a single request and its arguments [17]. Pagination refers to techniques for programmatically querying all pages to obtain the entire return dataset [17].

In total, we collected 53,414 tweets posted between April 2019 and December 2022 containing the terms “obese” and “obesity.” We specifically analyzed original tweets to understand public, celebrity, and organizational sentiments about obesity during the COVID-19 pandemic. We chose original tweets as they often originate key discussions and reflect the genuine stance of the users, particularly influential figures whose opinions can significantly sway public discourse. This allowed us to distill the core narratives and sentiments surrounding obesity, unobscured by the potential dilution or bias that retweets and comments might introduce. These latter elements, while undeniably valuable for gauging the spread and engagement with certain opinions, can encompass a wide array of responses, from supportive echoes to direct rebuttals or even tangential discussions.

### Data Preprocessing

Before conducting sentiment analysis and topic modeling, data preprocessing was performed to clean and normalize the collected tweets using *NeatText* package in Python [18]. This involved the following steps [18]:

1. Removing URLs, special characters, and numbers
2. Converting all text to lowercase
3. Tokenizing the tweets into individual words
4. Removing stop words (commonly used words that do not carry significant meaning)

### Celebrity Influence Analysis

Our methodology for assessing the influence of celebrities and important organizations involved analyzing the engagement metrics of their tweets related to obesity. We identified tweets from these individuals that resulted in a marked increase in Twitter activity, visualized as spikes in tweets on the topic of obesity over time. The events and tweets corresponding to these spikes were identified by the researchers (AW and SSA) from the Twitter records. This analysis provided insight into how celebrity statements on obesity resonated with and mobilized their audience, serving as an indirect yet insightful indicator of a tweet’s impact on public attitudes and, by extension, its potential public health implications.

### Sentiment Analysis Using Transformer Language Model XLM-RoBERTa-Base-Twitter

Our aim was to classify tweets into 3 sentiment classes: positive, negative, and neutral. To achieve this, we used zero-shot text classification, which allows for the categorization of tweets without explicitly training the model. The pretrained transformer language model XLM-RoBERTa-base-Twitter (XLM-Twitter) by Cardiff NLP was used for this purpose [15]. This model has been fine-tuned on approximately 198 million tweets for various multilingual and monolingual apps, including sentiment analysis in 8 different languages [15]. The model processes the input tweets and generates an output with 3 labels (positive, neutral, and negative), 3 probability scores, and the maximum value from the scores as a label for the input tensor. The probability scores are calculated using the *softmax* function, which converts the raw output values (logits) into probabilities [15].

### Topic Modeling Using BERTopic

In this study, we followed the original BERTopic framework, using BERT (Bidirectional Encoder Representations from Transformers) for document embeddings, UMAP (Uniform Manifold Approximation and Projection) for dimensionality reduction, and HDBSCAN (Hierarchical Density-Based Spatial Clustering of Applications with Noise) for clustering [16]. The process involves 4 main steps.

#### Mapping Words and Documents to Real-Value Vectors

BERT, a pretrained language model, is used to convert words and documents into meaningful real-value vectors. These embeddings capture semantic information, enabling the identification of similar words and documents [16].

#### Performing Dimensionality Reduction

UMAP is used to reduce the number of features of the embeddings while preserving the global structure of the vectors in a lower-dimensional space. UMAP is a manifold learning technique that uses concepts from differential geometry and algebraic topology. It has 2 main hyperparameters, the number of nearest neighbors (*$n$*) and the minimum distance (*$minDist$*), which control the balance between local and global structure preservation [16].

#### Clustering the Documents

HDBSCAN is used for clustering. This algorithm combines the advantages of hierarchical clustering methods with DBSCAN (Density-Based Spatial Clustering of Applications with Noise). It does not require previous knowledge of the ideal number of clusters and allows for the automatic recognition of noise data. HDBSCAN has 2 main hyperparameters, the minimum cluster size (*min_cluster_size*) and the minimum samples (*min_samples*), which control the density-based clustering process. HDBSCAN works by constructing a hierarchical tree of clusters and selecting the most stable clusters throughout the tree as the final result [16].

#### Retrieving the Most Representative Words for Each Cluster

A class-based variation of term frequency–inverse document frequency is used to identify the most representative words for each cluster [16]. Term frequency–inverse document frequency is a common weighting scheme that aims to provide a vector representation of each document, reflecting the importance of each word by calculating the normalized product of the term frequency and the inverse document frequency.

#### Spikes in Twitter Activity Over Time for Each Cluster

An analysis of Twitter response activity was performed with a focus, particularly on the spikes in public response toward different clusters. This involved examining the volume and velocity of engagement across the identified clusters, which helped us understand the public’s reaction intensity to various obesity-related discussions. By mapping these spikes in response activities to specific clusters, we were able to further elucidate the public’s concerns and interests regarding obesity during the COVID-19 pandemic, providing additional layers of context to our findings.

Finally, the 2 authors (AW and SSA) conducted a manual analysis for the top 20 clusters, for an in-depth understanding of the prevalent attitudes and perceptions regarding obesity among Twitter users. The top 20 clusters were assessed for homogeneity, and those representing similar broad themes were merged by the authors. Representative tweets were cited after anonymization, to corroborate authors’ interpretations of the clusters.

### Ethical Considerations

Our study exclusively analyzed publicly available tweets from Twitter. The data used in our analysis consist of information that was shared publicly by users on the platform, without any expectation of privacy for content posted in the public domain. Given the nature of our study, it falls under the category of research exempt from ethical review according to many institutional and national guidelines on research ethics, which typically do not require ethical approval for studies involving the analysis of publicly available, nonsensitive data where individuals cannot be identified.

## Results

### Sentiment Analysis for Tweets Regarding Obesity

**Figure 1:**
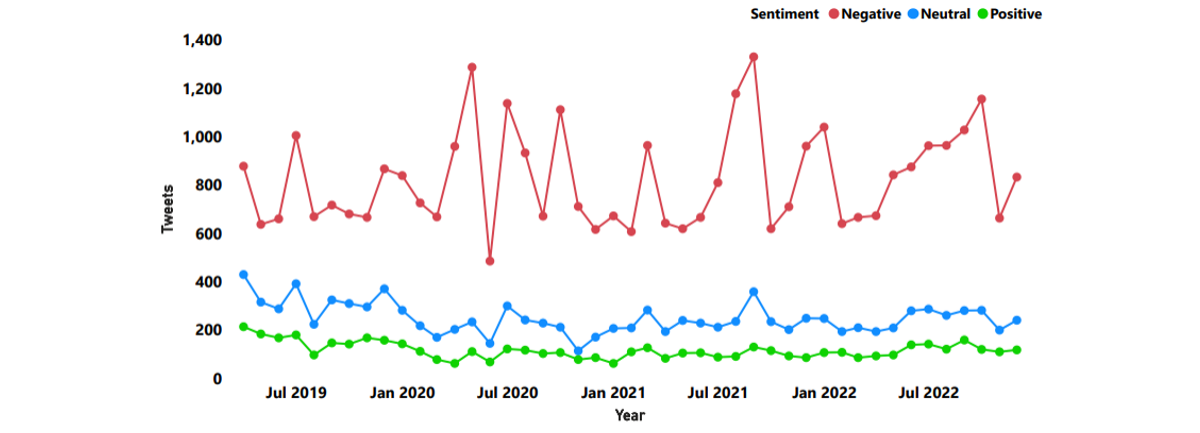
Volume of tweets on obesity and their breakdown according to sentiments (April 2019 to December 2022).

**Figure 2:**
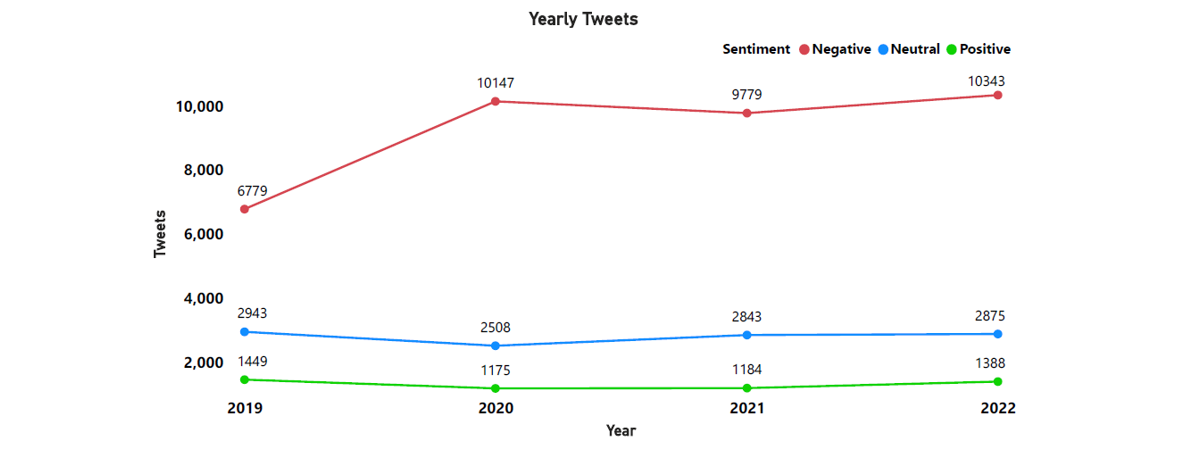
Sentiment-wise yearly trend in tweets regarding obesity (April 2019 to December 2022).

The Twitter API was used to extract 53,414 tweets posted between April 2019 and December 2022 containing the terms “obese” and “obesity.” Sentiment analysis revealed a substantially higher percentage of tweets (n=37,048, 69.36%) represented negative sentiments, followed by neutral (n=11,169, 20.91%) and positive (n=5196, 9.73%; Figure 1). A steadily increasing trend in negative sentiments was observed among Twitter users from April 2019 to July 2022, while the volume of neutral and positive tweets remained stagnant (Figure 2). Individual tweets classified according to their sentiments can be accessed (Multimedia Appendix 1).

Spikes in Twitter activity (Figure 1) were associated with significant political and public health events. Increased Twitter activity was noted pertaining to the link between increased hospitalizations among people with obesity [19]. On March 4, 2021, research findings on increased risk of death by COVID-19 among people with obesity were reported. Similarly, on April 21, 2020, the US Centers for Disease Control and Prevention (CDC) reported higher frequency of COVID-19–related deaths among people with obesity. Other public health–related events attracting increased Twitter activity included an obesity campaign initiated by the UK government, which drew a lot of criticism from the public (July 27, 2020).

In a similar manner, several tweets from political personalities resulted in spiked Twitter activity. On August 23, 2021, a US-based political commentator’s comments on the refusal to vaccinate people with obesity for COVID-19 sparked outrage. Increased Twitter activity was generated after a high-ranking official passed negative remarks on the US President’s struggle with obesity (May 19, 2020). On October 2, 2020, the US President’s diagnosis of COVID-19 also sparked negative sentiments toward obesity. Among celebrities associated with the entertainment industry, their struggle with obesity was also highlighted negatively (October 9, 2022).

### Topic Modeling

#### Overview

**Figure 3:**
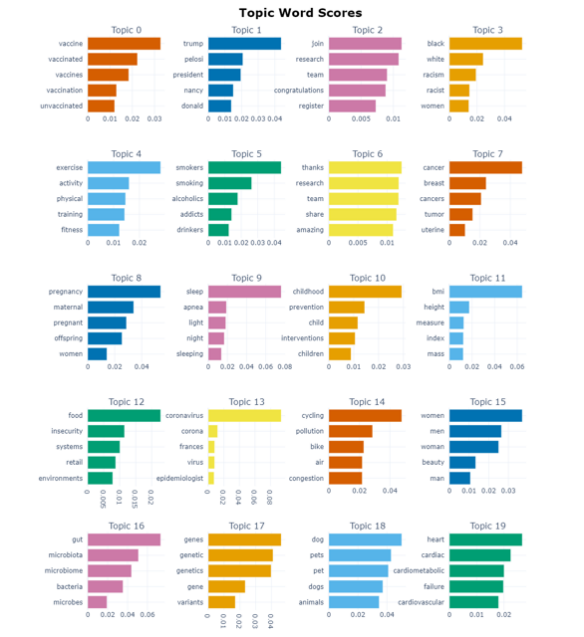
Popular topics of tweets regarding obesity.

**Figure 4:**
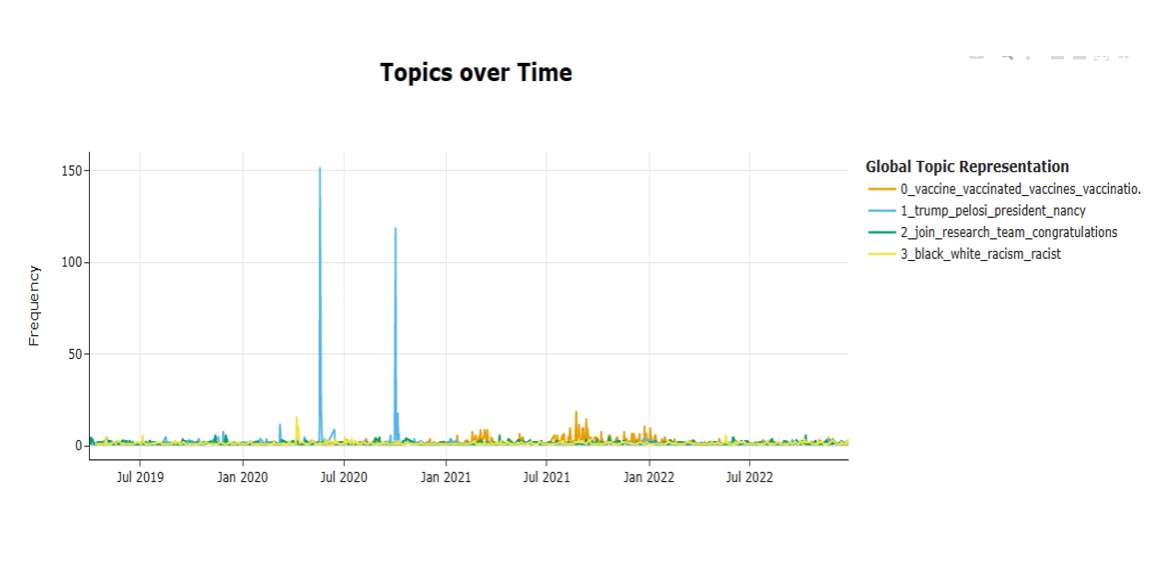
Frequency of tweets visualized over time for the 4 largest topic clusters.

**Figure 5:**
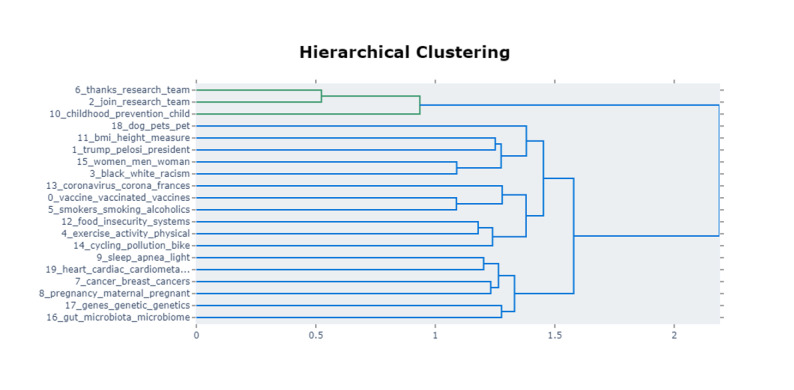
Dendrogram presenting clusters and subclusters of tweets regarding obesity during COVID-19 pandemic.

Topic modeling revealed 243 parsimonious clusters (Multimedia Appendix 2). These represented different clusters of tweets on obesity. For brevity, the top 20 clusters of keywords comprising the highest proportion of tweets are presented in Figure 3. An intertopic distance map for obesity-related tweets generated using BERTopic is provided in Multimedia Appendix 3. This map presents the density of each cluster and the relationships and relative distances between clusters generated through topic modeling. Among the 4 largest clusters (Figure 4), comments by the 52nd speaker of the United States House of Representatives on the US President’s struggle with obesity generated large spikes followed by a decrease in tweets. Cluster 3 on obesity and racism generated increased activity in 2020 and cluster 0 on vaccination for COVID-19 generated increased activity after January 2021. A line plot showing trend of tweets from 20 largest clusters is provided in Multimedia Appendix 3. The dendrogram presenting clustering of similar and dissimilar clusters of tweets is presented in Figure 5. A qualitative analysis of the top 20 clusters of tweets was performed by the authors (Table 1).

**Table 1:** Sample tweets pertaining to important clusters regarding obesity.

Cluster	Theme	Tweet
15	Body shaming	This woman was extremely morbidly obese and a proponent of “body positivity” and “fat acceptance.” She is now dead from obesity. She was literally killing herself on camera while being applauded. Fat acceptance is a murderous, suicidal ideology and should be called out as such. Fat acceptance is a death cult. It’s one of the most dangerous ideas in the world...It’s probably time that we start thinking about the misinformation that tells people...that morbid, suicidal, self-destructive obesity is something to be celebrated. Body positivity is not for obese people. It’s for burn victims. Stop trying to excuse yourself for unhealthy behavior.
1	US President’s struggle with obesity	Biden has Covid Is resting, no fuss because he is in shape and fully vaccinated When trump had covid was medivac to the hospital, had an army of medical people taking care of him because he is obese and ignored the vaccines. Being an idiot is costly to taxpayers
0 and 13	COVID-19 and obesity	And yet, as usual, this latest study ignores that obesity is largely driving COVID hospitalizations and deaths, which is relevant if we’re considering “all-cause health events.” @washingtonpost “New study”… so we’re just going to ignore the fact that every study since the start of the pandemic showed that obesity was the main (and often only) factor in struggling with Covid. If you weren’t really old or fat, you never had to worry. This is dumb. By May 2020 we knew Covid was a disease of the elderly and obese. We should have locked down via BMI and age. Delivered healthy meals to the elderly and diet meals for obese. Could have done some good for the world. 7. By waiting to get the vax, you’re burdening our healthcare system if you get Covid. The majority of ppl in hospitals w/ Covid have serious co-morbidities, which are the real strain on the system. Obesity kills almost as many ppl per yer as Covid has killed globally.
3	Racism	Black grifter playbook: Tell them their obesity, violent aggression, poor educational attainment & absent fathers are the result of factors outside of their control that they're not responsible for. Tell them they’re perfect as they are; blame everything on racism! Obesity is the number one comorbidity when it comes to COVID. The black community, especially black women in America are disproportionately obese. These are facts and this is why there is a higher death rate. But you push the racism angle.
4, 10, and 14	Physical activity, prevention, and interventions	I never saw what people think the issue with going to the gym bc you don’t want to be fat is, obesity is a massive issue around the world and can cause health issues that exercise and a good diet can help prevent lmao
2, 5, 7, 9, 14, 16, 17, and 19	Health research of obesity and its antecedents and consequences	Hadassah-University Medical Center researchers said they have found the mechanism that contributes to the aggressive progression of breast cancer in overweight women. It’s amazing that a lot of Americans think they are healthy. If you are overweight, obese, have high blood pressure, diabetes, sleep apnea, if you smoke, drink excessively, can’t walk a flight of stairs w/o getting short of breath, etc... you aren’t healthy. Gut microbiota alterations in obesity remain a subject of debate. These findings report a decrease in gut microbes associated with inflammation (undesirable Bacteroides 2 enterotype) in individuals with obesity associated with the use of statin drugs. In Brazil, 53% of men are obese. 45% of women are obese. High levels of cardiometabolic syndromes. They either fix their health or COVID will fix it for them. It’s not just about CO2. Biking is superior in every single way: congestion, road safety, public health (obesity, cardiovascular), air pollution, noise pollution (think animals, sleep quality), rents (think smaller roads, more housing) etc
10	Childhood obesity	Join @UNICEF and @WHO on World Children’s Day for a discussion on childhood obesity prevention and how to maintain momentum in the challenging new COVID-19 reality.
12	Food insecurity	Interventions aimed at making food retail environments health promoting require targeting a combination of individual, intrapersonal and environmental factors, research by our @GLOBE_obesity centre has found. UK #obesity plan will fail without action on unhealthy #food. Centre for Food Policy says efforts to lose weight are being thwarted by constant #advertising and exposure in retail.

#### Restricting Access to Vaccination for COVID-19 for People With Obesity

This domain was discussed in cluster 0 and 13, with a total of 1367 tweets. This cluster presented mainly negative perceptions of the public toward people with obesity. Themes surrounded restricting access of COVID-19 vaccination to people with obesity. This was mainly due to the public perception that obesity led to increased rates of COVID-19 hospitalization and deaths and that people with obesity were to be blamed for this.

#### US President’s Struggle with Obesity

This theme was predominant in cluster 1 with 1003 tweets.

#### Health Research on Obesity and Its Antecedents and Consequences

This theme was discussed in clusters 2, 7, 9, 16, 17, and 19, comprising a corpus of 2910 tweets. This theme focuses on the cardiometabolic consequences of obesity, genetic risk factors, links with gut microbiota, and the association of different cancers with obesity. Although the dominant theme remained research discussions, however, a small corpus of tweets in cluster 5 discussed the syndemic association of obesity with alcohol and smoking or cited obesity as a risk factor for different disorders along with alcohol and smoking. This suggests that obesity is being perceived and treated similarly to alcohol consumption and smoking, in terms of social stigmatization, health concerns, and public policy. Research on sleep apnea and links with obesity was also highlighted.

#### Racism and High Obesity Rates Among Black American Population

This was a predominant theme in cluster 3 with 583 tweets. This corpus of tweets was predominated by prejudice toward Black American people. Race was associated with negative perceptions such as high prevalence of criminality, aggression, poor education, and obesity among Black American population.

#### Glorifying and Body Shaming

This corpus of tweets comprised 295 tweets and was a predominant theme in cluster 15. It explored the dichotomy in beauty standards that can be observed in society, particularly with regard to the perception of obesity and being overweight. It highlighted the complex nature of beauty standards in modern societies, where there is a growing awareness of body diversity and inclusivity. Several tweets celebrated and promoted larger body sizes as desirable and attractive, often as a response to the pressure and stigma faced by people with larger bodies. On the other hand, large groups of Twitter users promoted body shaming based on societal norms and expectations.

#### Childhood Obesity

Childhood obesity was a predominant topic in cluster 10 with 338 tweets. It highlighted the celebration of World Children’s Day by the World Health Organization where UNICEF (United Nations International Children’s Emergency Fund) and the World Health Organization discussed childhood obesity prevention, especially during the COVID-19 period.

#### Food Insecurity

This was a predominant topic in cluster 12 with 329 tweets. These discussed studies pertaining to healthy food retail policies in Australia, and policies and issues pertaining to healthier food choices and ultra-processed food.

## Discussion

### Principal Findings

By combining the sentiment analysis and topic modeling results, we were able to gain insights into the public discourse on obesity as reflected by the collected tweets.

Our analyses regarding the power of social media in shaping the public discourse on obesity are corroborated by similar studies published recently. White et al [20] examined the role of social messaging shared by persons in the public eye, based on 13 million tweets published during the COVID-19 pandemic. They demonstrated that COVID-19–related tweets published during the pandemic held a more negative tone. Authors also found that tweets shared by celebrities shape risk perceptions, political ideologies, and health-protective behaviors, and thus, influence public sentiment and discourse direction [20].

Our analysis showed that obesity was a frequently discussed topic on Twitter throughout the COVID-19 pandemic. The number of tweets concerning obesity matched those observed in studies for asthma [21] and bowel cancer [22], albeit falling short of the volume seen in discussions about breast cancer [23]. Through a comprehensive analysis, we found that conversations around obesity were not only widespread but also dominated by notably negative sentiments. As the pandemic unfolded, tweets from influential celebrities, politicians, and organizations on obesity generated a lot of debate among the users, who took to Twitter to share their thoughts, experiences, and concerns regarding obesity. When celebrities posted negative comments or opinions about obesity, their followers were more likely to engage in similar conversations, further perpetuating the negativity. These negative tweets could have various implications, such as reinforcing stereotypes and stigmatizing individuals struggling with obesity [24-26]. The ripple effect of these celebrity-driven conversations might lead to increased self-consciousness, social isolation, and negative self-image among those dealing with weight-related issues [27]. This demonstrates the vital role of celebrities in shaping public opinion and discourse around health topics, leading to a profound public health impact.

### Exclusion of People With Obesity

Research has shown that obesity is associated with profound stigma, stereotypes, and implicit biases [27-29]. Our analyses revealed 2 important clusters of tweets demonstrating biases against people with obesity. These clusters of tweets revealed implicit health-related and racial biases toward people with obesity. This debate was generated after research insights were published on the role of obesity and ethnicity in worsening COVID-19–related outcomes [19]. The US CDC’s communication [19] regarding the risk of severe outcomes related to the COVID-19 pandemic among Black people and people with obesity received significant attention on Twitter. While CDC’s communication was important from a public health perspective, it revealed biases against obesity among celebrities and public. These implicit biases against obesity led to unempathetic notions like denying COVID-19 vaccinations to people with obesity because they are responsible for their own condition. A large cluster of tweets also discussed obesity and COVID-19 outcomes from the lens of racism, leading to emotionally charged debates. Due to these issues in the United States, Black people faced a lot of stigma and prejudice.

### Research and Intellectual Discourse on Obesity

In addition to its challenges, Twitter has proven to be an effective platform for intellectual discourse, fostering scientific communication and engagement. Scientists often published their research findings related to obesity, communicated with the public, and advertised their studies for recruitment. Twitter also allowed engagement with diverse audiences which resulted in real-time dissemination of scientific findings [30,31]. Moreover, such social media platforms also help in networking with other scientists, and foster collaboration within the scientific community [30,31]. Social media platforms like Twitter are also important media for exploring the public’s sentiments regarding health policies. Twitter could provide an important avenue for discussing health policies and help shape intellectual discourse on obesity. Our analyses demonstrated several useful insights regarding the UK government’s “Better Health” campaign [32].

### Implications for Future Research and Practice

This research has several implications. It is essential for celebrities to be mindful of their impact on public opinion and the potential consequences of their statements. A recent international consensus statement published by multiple stakeholders highlighted the stigma and prejudice against people with obesity [6]. It emphasized that both the general public and medical professionals have been reluctant to accept obesity as a disease and widespread beliefs persist that obesity is entirely within an individual’s control [33]. This leads to weight-based discrimination and unfair treatment at workplaces, health care settings, and educational institutes [6]. In health care environments, negative attitudes held by health care professionals regarding obesity, such as associating it with laziness, lack of willpower, and poor self-control, can negatively impact the quality of care provided to individuals with obesity [6]. Therefore, it is important to launch health information campaigns to promote knowledge regarding the complex nature of obesity and reduce stigma and prejudice against people with obesity [6,27]. Encouraging a more compassionate and supportive conversation about obesity can contribute to a healthier and more inclusive atmosphere, which is crucial for those dealing with the physical and mental struggles related to obesity.

### Limitations

This study has some limitations. Our analyses excluded comments and retweets, which constrains our ability to fully delve into the dynamics of public discourse on Twitter. Nevertheless, it afforded us the clarity and manageability necessary for an effective sentiment analysis and topic modeling, presenting a focused snapshot of sentiments during a pivotal time. Acknowledging the limitations of this approach, we propose that subsequent research endeavors could broaden the scope to include retweets and comments. Such expansions would enrich our understanding of the engagement dynamics and the propagation of sentiments within the diverse ecosystem of Twitter, offering a more comprehensive view of the public discourse on obesity.

Given our study’s aim to unravel public emotions and sentiments toward obesity during the COVID-19 pandemic, we opted for a more focused approach to ensure the relevance and coherence of our dataset. By limiting our analysis to “obese” and “obesity,” we were able to maintain a clear and specific thematic focus, which is crucial for the accuracy of sentiment analysis and topic modeling. We acknowledge that this decision narrows the breadth of our investigation and may exclude certain relevant discussions. However, this specificity was necessary to achieve our research objectives and ensure the integrity of our findings. Future studies could expand on this work by exploring a broader array of terms and using more sophisticated filtering techniques to capture a wider spectrum of public discourse on body weight and health.

The scope of our research was intentionally focused on analyzing public sentiments and topics related to obesity as reflected in original tweets. This focus inherently limited our exploration into the dynamics of Twitter, such as the impact of retweets and comments, which could offer additional layers of understanding regarding public engagement and sentiment propagation. Furthermore, while the influence of celebrities and political personalities on public opinion was noted, a detailed quantitative analysis of their tweets and the direct correlation with increased Twitter activity was beyond the scope of our current study. Such an analysis would require an extension of our dataset to include a broader range of tweets, potentially encompassing various user categories and their interactions. While these limitations constrain the breadth of our analysis, they also highlight areas for future research. Future studies, with access to expanded datasets and resources, could explore these dimensions more comprehensively.

Finally, future studies could benefit from incorporating advanced bot detection techniques to further refine the analysis of Twitter data, ensuring that the insights derived are truly representative of human sentiment and discourse on the topic of obesity.

### Conclusion

Twitter is an important source to gauge obesity-related sentiments and attitudes of the public, celebrities, and influential organizations. Sentiments regarding obesity were predominantly negative. Obesity was associated with racism, poorer life choices, and social evils such as illicit substance use and alcohol consumption. Influential politicians’ negative portrayal of obesity among their colleagues may lead to poorer public sentiments. This has negative connotations for public health in general. The Conservative government’s campaign for curbing the “epidemic” of obesity in Britain attracted criticism from the public. Obesity being a risk factor for severe COVID-19 also led to a negative portrayal on social media.

The study aims to contribute to a more comprehensive understanding of the role of social media in shaping public sentiment, attitudes, and health behaviors related to obesity. In addition, the findings will inform the development of evidence-based public health policies, prevention strategies, and treatment approaches that address the unique challenges posed by this global health issue in the context of social media and digital communication.

## Appendix

Multimedia Appendix 1 Sentiment analysis for individual tweets.

Multimedia Appendix 2 Results of cluster analysis.

Multimedia Appendix 3 Intertopic distance map and topics over time.

## Abbreviations

API: application programming interface
BERT: Bidirectional Encoder Representations from Transformers
CDC: Centers for Disease Control and Prevention
DBSCAN: Density-Based Spatial Clustering of Applications with Noise
HDBSCAN: Hierarchical Density-Based Spatial Clustering of Applications with Noise
UMAP: Uniform Manifold Approximation and Projection
UNICEF: United Nations International Children’s Emergency Fund

## Data Availability

All data are available for download using the Twitter application programming interface (API). The datasets generated during and/or analyzed during this study are available in the X developer platform [34].
